# Design and Prediction of Aptamers Assisted by In Silico Methods

**DOI:** 10.3390/biomedicines11020356

**Published:** 2023-01-26

**Authors:** Su Jin Lee, Junmin Cho, Byung-Hoon Lee, Donghwan Hwang, Jee-Woong Park

**Affiliations:** 1 Drug Manufacturing Center, Daegu-Gyeongbuk Medical Innovation Foundation (K-MEDI Hub), Daegu 41061, Republic of Korea; 2 Medical Device Development Center, Daegu-Gyeongbuk Medical Innovation Foundation (K-MEDI Hub), Daegu 41061, Republic of Korea

**Keywords:** in silico, aptamer, SELEX

## Abstract

An aptamer is a single-stranded DNA or RNA that binds to a specific target with high binding affinity. Aptamers are developed through the process of systematic evolution of ligands by exponential enrichment (SELEX), which is repeated to increase the binding power and specificity. However, the SELEX process is time-consuming, and the characterization of aptamer candidates selected through it requires additional effort. Here, we describe in silico methods in order to suggest the most efficient way to develop aptamers and minimize the laborious effort required to screen and optimise aptamers. We investigated several methods for the estimation of aptamer-target molecule binding through conformational structure prediction, molecular docking, and molecular dynamic simulation. In addition, examples of machine learning and deep learning technologies used to predict the binding of targets and ligands in the development of new drugs are introduced. This review will be helpful in the development and application of in silico aptamer screening and characterization.

## 1. Introduction

Aptamers are single-stranded nucleic acid substances with a high affinity for target substances [1,2]. Aptamers for a wide range of target substances, such as cells, viruses, protein, and even small molecules including toxic molecules, antibiotics, and hormones have been developed [3,4]. Aptamers have also been used as an alternative to antibodies because of their high specificity and binding ability to target substances. Compared to antibodies, aptamers can be flexibly changed in structure and are relatively small in size. Therefore, aptamers can recognize and bind to targets that are inaccessible to antibodies, such as targets with hidden binding domains. Moreover, aptamers can be mass-produced at low cost and are stable in most environments; thus, the application range is much wider. Another advantage of the aptamer is that it is limitless in its potential targets including toxic molecules or pesticides [4]. Based on these advantages, aptamers are widely used in diagnostics, medicines, cell imaging, biosensors, biochips, and drug delivery systems [5]. Aptamers are developed through an in vitro method called systematic evolution of ligands by exponential enrichment (SELEX), developed in the 1990s [1,2]. Through the SELEX process, which performs repeated binding with the target material, it is possible to specifically bind to the target material with high binding affinity. The process first starts with a random nucleic acid library containing 10^12^–10^14^ molecules. By reacting the random nucleic acid library with the target material, a conjugate between the target material and the nucleic acid is generated. The resulting target–nucleic acid conjugate is separated from the rest of the library that does not bind to the target. The specific sequences bound to the target are separated from the target and amplified through a method such as polymerase chain reaction (PCR). Amplified and single-stranded nucleic acids are repeatedly reacted with the target material. Generally, the SELEX process repeats the above process 6–15 times; however, it has the following difficulties. First, it may take several weeks to several months to discover aptamer candidates, and the success rate of development remains low. In addition, only a limited number of sequences among the aptamer candidate group can be synthesized and subjected to binding analysis with a target. 

A technique assisted by a computer is being developed [6] that predicts the binding affinity to the target material through the analysis of structural information [7]. Online-server-based programs such as RNAfold or RNAcomposer predict the secondary and tertiary structures of RNA/DNA [8]. This is because, as in the case of non-aptamer RNA/DNA, structural information can be obtained through the above server. It can be applied to the selection of aptamers that bind to proteins through molecular docking, providing molecular dynamic prediction based on structural information [9].

Artificial intelligence algorithms, such as machine learning/deep learning, are influencing computer-based aptamer selection methods [10]. Some machine learning techniques even outperform conventional molecular-docking-based binding-affinity prediction methods [11]. In addition, a small-molecule substance that induces miRNA–mRNA binding was developed using a binding affinity prediction tool based on artificial intelligence [12]. Although it has not yet been used in earnest for aptamer development, machine-learning/deep-learning-based methods will ultimately play a large role in the prediction of the binding of aptamers and target substances. As aptamer structural information is not required, numerous experimental data can be analyzed effectively. In addition, in this data analysis, training is conducted in reverse. Therefore, in this review, we examine various computer-based aptamer binding affinity prediction methods, such as machine learning/deep learning.

Research using in silico methods [13], such as molecular docking of bioinformatics and high-throughput SELEX (HTS) [14] in which next-generation sequencing (NGS) is applied to SELEX, is being conducted to increase efficacy. Recently, Yan et al. conducted a study on the scoring of nucleic acid–ligand binding [15].

In silico aptamer design mainly uses molecular binding technology. The quantitative structure–activity relationship (QSAR) method, which is widely used in drug design, is also used in aptamer design [16].

Aptamers can be designed that bind to complex polymers such as small molecules or proteins. However, with the current technology level, it is impossible to design aptamers that bind to cells. Using a molecular modelling method, it is possible to identify structural patterns important for the binding between an aptamer and a target substance, and through this, it is possible to enhance the binding affinity. This is because there are cases in which point mutations help the binding affinity between nucleic acids and target substances.

**Figure 1:**
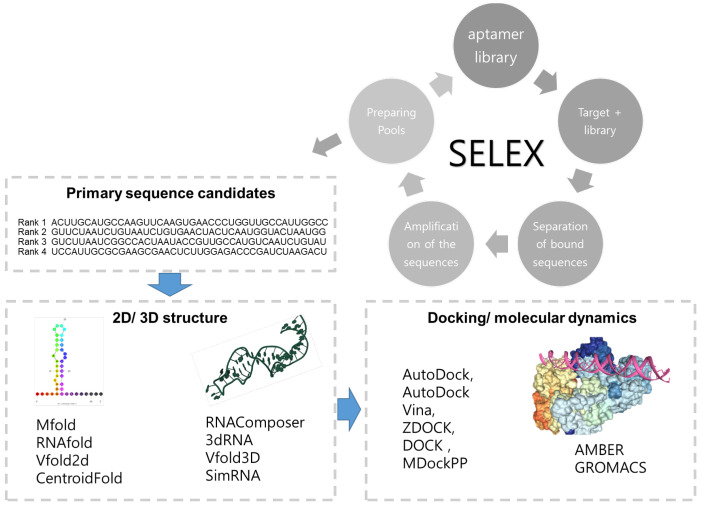
In silico design of aptamer.

Figure 1 is a typical flow of in silico aptamer design. The design starts with secondary structure prediction, proceeds with tertiary structure optimization, and then structural docking simulation is performed between the target material and the aptamer candidate group. In this process, the candidate group with the lowest energy is selected. In addition, molecular dynamics simulation can be performed to measure the stability and binding energy between the aptamer and target substance. Afterwards, the binding affinity between the aptamer and the target is analysed, and the binding affinity can be increased by applying point mutation or chemical modification. Through the repetition of this method, the binding affinity of the aptamer can be increased.

## 2. Prediction of the Aptamer Based on Its Structure

Recently, computer-based methods for selecting aptamers through aptamer structure prediction have been developed, and convenient and accurate aptamer development methods have been studied. The development method consists of four major steps [17]. First, the secondary structure of the aptamer is predicted based on the sequence; then, the tertiary structure is predicted and integrated with the secondary structure. Subsequently, molecular docking is performed to predict the structure of the aptamer and target material. Finally, the stability between the aptamer and target material is evaluated and analysed through dynamic simulation.

### 2.1. 2D Structure Prediction of Aptamers

The secondary structure of an aptamer plays a key role in binding to a target substance [18]. For example, it is known that the bonding strength increases when secondary structures such as hairpin structures, G-quadruplexes, and T-junctions are formed [19]. The secondary structure is also highly related to the prediction of tertiary structure [20]. In this regard, various computer algorithms have been developed and used to predict the secondary structure of aptamers.

The computer-based prediction principle is similar for both DNA and RNA aptamers. Secondary structure prediction algorithms can be classified into two types: the free energy analysis method and nucleic acid sequence configuration analysis method [21]. RNAfold predicts the secondary structure based on the minimum free energy, given a nucleic acid sequence. Another free-energy-based prediction method is Mfold [22]. RNAfold has been utilised for the development of tetracycline aptamers [23]. 

The RNAstructure online web server, which uses the free energy minimisation method first reported in 1998, has the maximum expected accuracy [24], stochastic sampling [25], and pseudoknot prediction [26], which have been extended to include multiple structural prediction techniques. The secondary structure of DNA aptamer binding to 17β-estradiol was predicted using RNAstructure [27]. An aptamer that binds to prostate-specific membrane antigen (PSMA) was developed using an aptamer structural analysis using RNAstructure and a protein/RNA docking analysis algorithm [28].

Vfold2d is a free-energy-based program that calculates loop energies of RNA motifs to predict the secondary structure of RNA [29]. 

The CentroidFold online web server uses a sequence alignment analysis method, and by arranging multiple RNA sequences, the secondary structure of overlapping regions can be predicted [30]. 

### 2.2. 3D Structure Prediction of Aptamers

Aptamers bind to specific targets to form complexes and can perform various physiological functions. As the tertiary structure determines the function of a biological molecule, accurate modelling of the tertiary structure is of the utmost importance. Recently, four online servers, RNAComposer, 3dRNA, Vfold3D, and SimRNA, which can predict the tertiary structure of RNA aptamers, have been developed. RNAComposer, 3dRNA, and Vfold3D make predictions through fragment analysis, whereas SimRNA is an energy-based prediction method. The tertiary structure can be predicted by entering the RNA sequence into the above server or by entering the secondary structure, applying the dot-bracket notation used to express the secondary structure [31].

In RNAcomposer, the input secondary structure is fragmented and matched with elements constituting the tertiary structure. RNAcomposer arranges the corresponding elements and assembles them into a complete three-dimensional structure. Among the completed three-dimensional structures, the structure with the lowest energy is used as the final three-dimensional structure of the RNA aptamer.

Hu et al. selected RNA aptamers for angiopoietin-2 by predicting the three-dimensional structure of RNA aptamers using RNAComposer [32]. 3dRNA, a tertiary structure prediction tool using another fragmentation technique, predicts the tertiary structure by utilising helices and loops of secondary structure elements [33]. This method predicts the final structure by assembling the templates after obtaining the tertiary template corresponding to the secondary structural element. Using 3dRNA, the tertiary structure of RNA aptamers binding to membrane proteins of *Streptococcus agalactiae* was predicted [34]. Similarly, Vfold3D identifies motifs such as helices and loops in secondary structures and retrieves optimised templates for each motif [29]. The tertiary structure of the final RNA aptamer is determined based on the structure with the smallest energy value by assembling the searched templates and calculating the energy for each structure. The tertiary structure of RNA aptamer binding to PSMA was calculated using the Vfold3D online web server [35]. SimRNA, which analyses the structure of RNA, simplifies nucleic acid chains in a coarse-grain method that removes other atomic parts while leaving atoms that exhibit RNA characteristics essential for binding in RNA chains. The program also uses data to analyse the energy needed to produce a stable three-dimensional structure [36]. The three-dimensional structure of the RNA aptamer that binds to angiopoietin-2 was designed using the SimRNA web server [35].

A comparison of the tertiary structure modelling software described above demonstrates that all software showed a similar level of accuracy for sequences less than 40 bp but significantly lower accuracy for tertiary structure analysis of relatively long sequences of 83 bp [37]. This was found by comparing five sequences, and although more data analysis is required, Vfold3D showed generally consistent scores and the smallest deviation. Thus, Vfold3D should be considered the most preferrable tool for tertiary structure prediction [37].

Although DNA aptamers are widely researched and developed, the number of DNA prediction models for tertiary structures is small compared to that for RNA [19]. RNA tertiary structure prediction techniques can also be used to predict the structure of DNA. The tertiary structure of DNA can be predicted by first generating the tertiary structure of RNA using RNAComposer and then transforming it into a DNA structure [38,39].

The treatment method proposed by Iman et al. can be largely separated into four processes [20]. First, the secondary structure of the DNA aptamer is predicted using the Mfold online web server and then converted into the RNA tertiary structure using Assemble2/Chimera software. Next, the RNA tertiary structure is converted back to the DNA tertiary structure using Visual Molecular Dynamics (VMD) software. Finally, the tertiary structure of the DNA aptamer is refined using VMD software. When this processing method was applied to 24 DNA aptamers with known tertiary structures, it was confirmed that the model-predicted tertiary structure matched the actual tertiary structure well.

### 2.3. G4 Structure of Aptamers

G-Quadruplex (G4) is a nucleic acid structure formed by guanine bases [40], in which Hoogsteen hydrogen bonds reside between four guanines. Aptamers containing many guanines are known to recognise various proteins by forming the G4 structure [41].

Aptamers with a G4 structure are used as anticoagulants and anticancer drugs because they have thermal and chemical stability and are resistant to nucleases in serum [42].

Structural analysis techniques such as nuclear magnetic resonance [43] or X-ray [44] are used to characterise the G4 structure; however, they are difficult to apply to many G4 structures. However, in silico techniques can identify G4 structures throughout the genome [45].

Prediction of G4 formation using DNA/RNA sequences is possible through scoring methods and machine learning methods, and qsfinder is known to be the most excellent among many G4 prediction programs [45,46].

Basically, pqsfinder provides a scoring function trained on G4-seq experimental data. The novel aspect accounts for the competition between them by estimating the total number of possible local structures, which outperforms competing tools by allowing for imperfections through the creation and training of advanced scoring models with improved accuracy compared to similar tools. Additionally, this new tool evaluates all competing forms and, using advanced options, provides customisation capabilities that can be easily and quickly extended or modified for future newly discovered rules and scoring functions [46].

### 2.4. Molecular Docking

Molecular docking is the most important tool for predicting optimal binding sites between proteins and ligands. For molecular docking, all possible binding sites between proteins and ligands must be searched and evaluated through the respective scoring of these binding sites [47]. AutoDock, AutoDock Vina, ZDOCK, DOCK and MDockPP are known to be useful for aptamer development.

AutoDock consists of AutoDock4 and AutoDock Vina [48]. AutoDock4 calculates the free energy to score binding sites, whereas AutoDock Vina scores binding sites using an empirical scoring function [49]. The empirical scoring function is a model trained on known binding affinity data of protein–ligand complexes. The binding energy is separated into energy items such as hydrogen bonding, ionic interaction, hydrophobic effect, and binding entropy and is evaluated through the training process in which the coefficient for each energy item is determined to be multiplied by each energy item.

AutoDock4 is suitable for hydrophobic, non-polar pockets, whereas AutoDock Vina is suitable for polar binding pockets [50]. Therefore, the molecular docking process of aptamers binding to angiopoietin-2 was performed using AutoDock Vina [51].

ZDOCK uses a fast Fourier transform (FFT) algorithm to search for bonding sites and scores by combining the shapes and electromagnetic properties of the bonding sites [52]. ZDOCK predicted molecular docking with greater than 70% accuracy in inter-protein binding [53]. 

DOCK performs binding site prediction based on the shape of a molecule [54]. Bonding sites are scored using Assisted Model Building with Energy Refinement (AMBER), a scoring algorithm that considers factors such as electromagnetic properties, van der Waals force, interatomic coupling, and angle [55]. A cytochrome p450 aptamer was developed using DOCK [56].

MDockPP also utilises an FFT-based docking algorithm [57]. MDockPP collects all estimated binding sites and then selects them using a scoring function based on existing data. MDockPP was used to predict the molecular docking of aptamers that bind to PSMA [28].

### 2.5. Molecular Dynamics 

After the molecular docking process, molecular dynamics (MD) simulation should be performed to evaluate the stability of the aptamer and protein complex and calculate the binding energy [17]. A typical MD process involves a simulation based on an original molecular setup involving interatomic reactions and a recording of the simulation process. This MD process takes significant time and resources because it must perform numerous treatments for millions of particles [58].

Recently, many relevant software programs such as AMBER [59] and GROMACS [60] include MD functions. The binding energy between a protein and an aptamer conjugate can be calculated simply by subtracting the sum of the energies of the protein and the aptamer from the energy of the conjugate [61].

### 2.6. Others Affecting Affinity

In addition to the nucleic acid sequence, factors that affect the binding ability of an aptamer to a specific target material include the composition of a buffer or the presence or absence of other aptamers. For example, Tris/K^+^ buffer helps form the G4 structure and enhances the binding affinity between the aptamer and target material, whereas PBS/Mg2+ destabilises the structure of G4 and is known to inhibit binding to the target material [62].

Metal ions are also known to affect G4 structure formation and inhibit the activity of aptamers [63]. Troisi et al. reported that when the G4 aptamer binds to thrombin, the binding affinity of other thrombin aptamers that bind to other binding sites increases [63].

Thus far, we have discussed tools that can predict MD, molecular docking, 2D structure, and 3D structure. Next, we examine the cases in which the introduced tools were used to develop aptamers.

## 3. Application of an In Silico Method for the Development of the Aptamer 

### 3.1. Aptamers Binding Proteins

Proteins are most widely used in aptamer research. Depending on the protein structure and function, the aptamer structure and design are different. In silico design enhances the binding affinity and focuses on the stability of the aptamer. In many studies, the in silico technique is applied to theoretical research or theoretical experimental research to improve the binding affinity of aptamers. Another objective of the study is to understand the structural patterns that play an important role in the binding of aptamers to proteins.

The most frequently used target for in silico aptamer design is thrombin. Because the inhibition of thrombin plays an important role in blood coagulation, it is used as an important protein as a target. The HIV1 protein is important because it is involved in the invasion of DNA viruses. In addition to this, because the epithelial cell adhesion molecule (EpCAM) is important as a tumour marker, aptamer research on the relationship between aptamers and EpCAM is also being actively conducted.

#### 3.1.1. Thrombin Binding Aptamers (TBA)

As thrombin converts water-soluble fibrinogen into water-soluble fibrin, the enzymatic activation of thrombin can be controlled to regulate blood coagulation. Thrombin-binding aptamers have been utilised to inhibit the activity of thrombin and are the most well-studied for in silico methods for designing aptamers.

Tseng et al. designed an aptamer that binds to thrombin using an entropic-fragment-based approach (EFBA) [64]. EFBA is utilised to determine the probability distribution of nucleic acid sequences in response to a target protein. It also defines the sequence and tertiary structure. EFBA is used to determine the degree of dispersion of nucleic acids and the length of the sequence. Molecular dynamic simulations can be performed using Amber10 software, and binding energies can be calculated using the molecular mechanics Poisson–Boltzmann surface area/generalised Born surface area (MM-PBSA/GBSA) algorithm [61]. The energy between the aptamer and thrombin obtained through the SELEX experiment is higher than that between the EFBA aptamer and thrombin.

Thrombin has two epitopes that can structurally bind to two aptamers [65]; thus, a number of DNA aptamers that bind to thrombin have been studied [41]. The tertiary structure of an aptamer can be predicted using PyMOL and 3DNA [66]. The protein structure can be predicted through PyMOL, and the nucleic acid structure can be predicted through 3DNA. Molecular dynamic simulation can be performed through GROMACS [58,60].

Varizhuk et al. synthesised a conventional thrombin-binding aptamer into a triazole-linked nucleoside and demonstrated that the binding affinity was improved compared to the existing aptamer [67]. In this process, molecular dynamic simulation through Amber 8 confirmed that Arg 70 and Arg 73 are involved in the formation of hydrogen bonds. Varizhuk et al. reported in another study that TBA15 modified with 5-nitroindole also increased its binding ability to thrombin [68]. The energy of the aptamer was calculated through the MM-GBSA method, MD research was performed using the Amber 10 package, and docking was analysed through Autodock 4.2.

Mahmood et al. immobilised the thrombin-binding aptamer on the nanopore and simulated the passage time, flow rate, and detection frequency [69]. The stability of the DNA structure at the molecular level fixed inside the nanopore and the stability of the DNA structure under electric fields of various strengths were tested. In addition, the degree of protein passing through the nanopore was simulated depending on the DNA structure. At this time, molecules such as proteins were used in the nanoscale molecular dynamics (NAMD) program [69].

Rangnekar et al. used weave tile as a linker for an existing thrombin aptamer to design a structure that binds to thrombin and utilised the AmberTools suite and Curves+ for the weave tile [70]. By connecting existing aptamers with a weave tile, it was possible to create a DNA structure that can increase the anticoagulant effect by 7 to 16 times.

Riesen et al. applied 8-aryl-guanine to G4 and the central TGT loop [71]. Through molecular dynamic analysis using Amber 12‘s ANTECHAMBER module, we studied whether internal 8-aryl-guanine modification is a reaction between the DNA base and amino acid of thrombin. Through the modification of 8-aryl-guanine, the most stable G4 and the highest binding affinity were confirmed.

Through molecular design elements described above, such as truncation and chemical modification, the binding affinity of the thrombin-binding aptamer was enhanced, and the anticoagulant effect could be controlled.

#### 3.1.2. Infectious Disease Marker Binding Aptamers

There are many different methods of testing using antibodies in laboratory tests of infectious diseases, and the aptamer has been in the spotlight as an alternative to antibodies since it was first introduced in 1990 due to its high binding force (nM-pM) to be compared to antibodies. In addition, aptamers can be used in various detection methods to identify causative pathogens for specific infectious diseases by specifically binding to bacteria or viruses that cause infectious diseases, and many advantages over antibodies such as ease of production and chemical modification, small size, reusable, high-temperature stability, and low production cost. In this section, in silico methods for the development of aptamers are introduced.

Sgobba et al. analysed aptamers for HIV integrase through molecular docking and molecular dynamics [72]. The previously developed 93del aptamer and HIV1 integrase were docked using the HEX program. In addition, the electrostatic response was calculated, and the molecular dynamics of the aptamer and HIV1 integrase complex were studied.

Nguyen et al. predicted the binding of proteins and aptamers by combining in silico modelling techniques and NMR spectroscopy [73]. The secondary structure of the aptamer binding to HIV1 reverse transcriptase (HIV1 RT) was predicted through NMR and the free energy calculation of Vfold2d. IsRNA was used for coarse-grained MD, and the candidate group with the lowest energy was designated and docking of the protein and aptamer was performed using the MDockPP [74] program.

P. Kumar and A. Kumar designed an aptamer that binds to influenza hemagglutinin using the Monte Carlo method [16]. Here, 98 databases were analysed using the QSAR Monte Carlo method and compared with the previously reported pIC50 [75] and structural parameters of aptamers. This shows that the QSAR Monte Carlo method can be used for aptamer design.

Song et al. developed an aptamer that binds to the receptor-binding domain (RBD) of the SARS-Cov-2 spike glycoprotein by combining the in silico method using a machine learning screening algorithm with the in vitro SELEX method [76]. SELEX DNA pool was analysed using SMART-Aptamer [77], and the binding of the aptamer and RBD was studied using molecular docking and molecular dynamics. Gupta et al. developed an aptamer that binds to the spike trimer antigen of SARS-CoV-2 with RNAfold for the prediction of the secondary structure and G-quadruplex [78].

Sabri et al. developed an aptamer against the anti-hepatitis B surface antigen [38] through docking and MD. Three previously reported aptamers were cut into five short aptamers, and the secondary structure through Mfold and tertiary structure analysis were performed using RNAComposer. The binding affinity was analysed using the MM-PBSA algorithm. As a result of the analysis, it was possible to confirm the region required for binding with the aptamer.

Soon et al. cut an existing 56-mer aptamer that binds to the *Streptoccocus agalactiae* surface protein (PDB ID 2XTL) and analysed its secondary structure using Mfold [34]. The 2D structure was transformed into a 3D structure using the 3dRNA 2.0 web server [33]. Afterwards, the RNA aptamer was docked using AutoDock Vina, and the binding affinity was analysed. Consequently, a 40-mer aptamer with excellent binding ability could be selected.

#### 3.1.3. Cancer Marker Binding Aptamers

In recent years, lots of aptamers for cancer treatments have been reported. Aptamers against various cancer biomarkers can be used for both the discovery of biomarkers and diagnostic and therapeutic purposes. Rockey et al. selected a 41-mer aptamer that binds to PSMA, using tertiary structure prediction and protein/RNA docking [28]. The secondary structure of the RNA aptamer was predicted using RNAstructure 4.6 [79], and a small aptamer with a similar binding ability to the existing aptamer was found using “rational truncation” technology. Through docking using Amber and MDockPP, the position of the aptamer involved in binding to PSMA was found.

Bavi et al. designed a 15-mer RNA aptamer that binds to EpCAM [80]. The secondary structure of RNA was obtained using the RNA Vienna program [81], and the secondary structure was converted into a tertiary structure using Rosetta software. Molecular dynamics were performed using Amber, and the binding free energies of the aptamers and EpCAM were calculated using the MM-PBSA method.

Bell et al. optimised the binding mode of the previously reported EP23 aptamer through docking with MD [82]. After predicting the structure of the RNA aptamer through Mfold, MD simulation was performed using NAMD 2 [83]. Subsequently, a docking study using Dot 2.0 [84] was performed. MD simulation was performed for 10 candidate groups selected by docking. Subsequently, an actual experiment using isothermal titration calorimetry was performed, whereby the two aptamers were reported to have higher binding affinity than the previously reported EP23.

Wang et al. studied aptamers that bind to carcinoembryonic antigen [39]. After creating a variant by adding or deleting bases to the previously reported aptamer sequence, the secondary structure was predicted through Mfold, the tertiary structure was predicted through the RNAComposer web server, and docking was performed using ZDOCK [52]. Afterwards, it was confirmed that the performance of the aptamer was improved by an experimental method. These studies show that the in silico post-SELEX screening method is meaningful in improving the performance of aptamers.

Santini et al. studied an aptamer for the transmembrane glycoprotein mucin 1 (MUC1) [85]. First, MD simulation using Amber 16 was performed to analyse the binding between the previously reported S2.2 MUC1 aptamer variant and the APDTRPAPG epitope of MUC1. The binding affinity between MUC1 and the aptamer was calculated through the MM-GBSA binding energy calculation. As a result of the study, it was reported that the new MUC1 aptamer containing T11 and T12 mutations more stably binds to the APDTRPAPG epitope.

Shcherbinin et al. developed an aptamer for cytochrome P450 using MD [56], and Zavyalova et al. developed a DNA aptamer that binds to thrombin using the MD of GROMAC4.0 [40].

#### 3.1.4. Other Protein-Binding Aptamers

Hu et al. performed a simulation of an aptamer binding to angiopoietin-2 (Ang2) using ZDOCK and the ZRANK docking function included in Discover Studio 3.5 [32]. By truncating 16 previously reported RNA candidates, the secondary structure was predicted through the CentroidFold web server [30], and the three-dimensional structure was predicted through the RNAComposer web server. Consequently, the aptamer with the highest binding affinity was reported to be 2.2 nM.

Cataldo et al. designed an aptamer that binds to Ang2 by structural prediction and molecular docking [51]. To this end, a number of variants of previously reported aptamers were generated and docked to the Ang2 protein. Then, the characteristics of the conjugate between the Ang2 protein and the aptamer were analysed. SimRNA [36] software was used for tertiary structure prediction. For docking, AutoDock Vina was used. The effective affinity was derived through the sum of the total energies, with the result of the effective affinity consistent with the experimental results.

Shcherbinin et al. developed an aptamer against cytochrome p450 [56]. A 15-mer aptamer was determined through docking and MD using the molecule DOCK 6.5 [86], and the binding energy of the aptamer and protein complex was calculated through MM-PBSA. After a tentative binding site was designated by performing docking between short sequences consisting of three bases and the cavity of the protein, aptamer candidates were identified by designating a sequence with low binding energy to the binding site. The binding affinity of the aptamer developed by the in silico method was 10^−6^–10^−7^ M.

Ahirwar et al. studied aptamers for oestrogen receptor alpha (ERα) [9]. ERα docking was performed using AutoDock Vina [48], Haddock [87], and PatchDock [88] using 18 sequences of human oestrogen response elements controlled by the oestrogen receptor as candidate groups. The binding affinity between ERα and the aptamer was predicted by measuring H-bonds and hydrophobic interactions between molecules. The stronger the H-bond is, the stronger the binding affinity of the aptamer is. The specificity of the selected 17-mer aptameter was determined experimentally.

Heiat et al. developed an aptamer by performing in silico analysis after SELEX to screen an aptamer for angiotensin II [89]. The secondary structure of the aptamer was predicted using Mfold 3.1 [22], and the tertiary structure was modelled using RNAcomposer. Molecular docking was performed using ZDOCK 3.0. The performance of the final aptamer was analysed experimentally.

Rabal et al. performed SELEX and in silico methods to design an aptamer that binds to the murine T-cell immunoglobulin mucin-3 (TIM-3) [90]. The tertiary structure was predicted using Rosetta [91], and docking of the aptamer and TIM-3 was performed using 3dRPC [92].

### 3.2. Aptamers Binding Small Molecules

For analyzing the small molecules with high accuracy and specificity, conventional methods such as HPLC and mass spectrometry have been used. However, the assay based on conventional equipment requires laborious pre-treatment and well-trained experts with high cost and space. On the other hand, a small sensing platform can be an alternative way with aptamers due to its limitless target range including toxic small molecules. In this section, well-established in silico methods for the identification of binding pockets with the prediction of structure, docking, and molecular dynamics are described.

Trinh et al. developed aptamers that specifically bind to fipronil, which is a widely used insecticide [93]. The developed aptamer was further studied with 3D molecular modeling for revealing the binding pocket. The 3D structure of ssDNA predicted by RNAComposer was loaded to discovery studio (DS) v18 to modify the structure of DNA. The refined ssDNA structure with GROMACS was studied with Lib-Dock module in DS for molecular docking. The binding site for the aptamer was decided to be the nucleotides from 5–11 and 20–26 with this 3D modeling technique.

Kadam et al. studied an aptamer against malathion, which is small toxic contaminant [94]. After SELEX, 4 candidates were selected based on the number of copies and the aptamer aptamers MalA1 and MalA2 were analyzed with in silico methods. Firstly, the secondary structure was predicted using Mfold and the dot-bracket confirmation was loaded to the RNAcomposer. The RNA PDB structure was visualized in DS v19 and then MD simulation was performed by GROMACs. Finally, the docking study was performed with Patch Dock and AutoDock vina for the identification of binding sites. 

In silico methods are mostly used for the analysis of the data from the HT-SELEX, truncation of the parent sequence, or identification of the binding pocket. For enhancing the binding affinity, the mutation of a specific region or truncation of the parent sequence is performed. Table 1 shows examples of direct comparison of affinity in terms of the dissociation constant.

Here, we have examined cases in which various in silico methods such as molecular docking and MD were utilised in various ways. Next, we investigate the use of machine learning/deep learning, a technology that has recently garnered attention for aptamer development.

## 4. Machine/Deep Learning for Designing the Aptamer 

Deep learning, as one of the machine learning techniques, learns and analyses data by simulating deep networks such as the human brain. Machine learning uses knowledge extracted from data and strengthens the internal relationship of data [98]. Machine/deep learning methods can be directly and efficiently used to predict massive sequences of next-generation sequencing data and can predict avidity more accurately. It can be accomplished by scanning and predicting the affinities of multiple sequences to one target simultaneously using structure-based methods.

A review of the research involving the identification of aptamers with high binding affinity through machine learning and deep learning is as follows.

### 4.1. Clustering for the Development of Aptamers Based on Machine Learning

Machine learning methods can be classified as feature-based and similarity-based methods. Feature-based methods use descriptors to create feature vectors, whereas similarity-based approaches use the "guilt by association" rule.

This rule starts with the assumption that similar drugs tend to interact with similar targets while similar targets are targeted by similar drugs [10]. An artificial intelligence/machine learning approach to predict the binding affinity of avidity was used to develop the state-of-the-art avidity prediction methods KronRLS [99] and SimBoost [100]. The binding affinity between the aptamer candidate and target is predicted based on the similarity between the candidates, which is generally evaluated by sequence- or structure-based clustering analysis. In Table 2, in silico tools for the design of aptamers are listed.

#### 4.1.1. Sequence-Based Clustering

The sequence clustering tool focuses on the similarities of the sequence consisting of A, T, G and C of the aptamer candidates in the pool. The tools use highly efficient algorithms for interpreting the sequence as the simple characters. AptaCluster calculates the similarity of the sequences of candidates based on the local sensitive hashing that categorizes similar strings into the same groups with high probability [108]. Both FASTAaptamer and PATTERNITY-Seq cluster sequences use Levenshtein distance [6,109]. The Levenshtein distance is determined by calculating the minimum number of insertions/deletions/replacements required to transform one word into another. AptaSUITE performs a framework analysis of data from HT-SELEX such as sequences and aptamer counts [102]. Since only A/T/G/C strings are used to represent aptamers, these sequence clustering models can analyse large SELEX data sets at high speed. Li et al. developed a web server named PPAI for the prediction of aptamers and the interaction between the protein and aptamer based on key sequence features of proteins and aptamers [104]. The PPAI integrates a machine learning framework of AdaBoost and random forest.

#### 4.1.2. Structure-Based Clustering

The structure-based clustering model predicts the binding affinity by comparing the structural motif of candidates and that of known aptamers for specific targets. Well-known examples of structure-based clustering models include AptaTrace and APTANI [110,111]. AptaTrace predicts the structural motif of the aptamer, and as the round progresses, sequences with overlapping structural motifs are sequenced. APTANI is a tool for analysing SELEX data based on the AptaMotif algorithm [111]. AptaMotif efficiently extracts structural motifs from SELEX-derived aptamers by an ensemble-based method. Caroli et al. reported APTANI^2^, which ranks aptamers with the information of the frequency and structural stability of each secondary structure predicted [106]. APTANI^2^ provides a graphical user interface that enhances its usability. SMART-Aptamer analyses the multilevel structure with unsupervised machine learning. The model finds the motif for binding with the consideration of the whole secondary structure [77]. RaptRanker first determines unique sequences in the data set, and all the subsequences of the unique sequences are clustered using the secondary structure features of the sequences [103].

The frequency of the subsequence cluster is used for the calculation of the average motif enrichment score, which is then applied for ranking the unique sequences. However, this model takes a long time to run because it predicts the secondary structure for each subsequence, and because it is based on clustering that can be biased toward aptamers that are very similar to sequences already observed, it limits the ability to optimise the SELEX results.

As an alternative meta-analysis platform, Shieh et al. developed AptCompare [105], which is a cross-platform program. AptCompare combines the most widely used analytical approaches for the identification of RNA aptamer motifs. The results can be obtained with the same GUI-enabled environment. 

### 4.2. Machine/Deep Learning for the Prediction of the Structure of Aptamers

#### 4.2.1. Machine/Deep Learning for Prediction of 2D Structure

Recently, research has been conducted to use machine learning techniques to predict the secondary structure of RNA, such as KNetfold or SPOT-RNA, to predict the secondary structure of aptamers. KNetfold predicts overlapping secondary structures by arranging RNA sequences as a hierarchical network of k-nearest neighbour classifiers [112]. KNetfold is an improved technique compared to existing secondary structure prediction tools, such as PFOLD or RNAalifold.

SPOT-RNA was designed based on two-dimensional deep neural networks and transfer learning [113]. Initially, models of ResNets and LSTM networks are trained on bpRNA datasets. The bpRNA data set is a data set of over 10,000 non-redundant RNA sequences with annotated secondary structures. The data sets generated by bpRNA are sufficiently large to train and test machine learning algorithms for RNA structure prediction, and detailed structural annotations provide the information needed to build useful and rich databases for RNA research.

The bpRNA is a novel annotation tool for RNA structures that includes complex pseudoknots. Previous studies analysing RNA structural topology from base pairs did not handle pseudoknots; however, bpRNA accurately generated dot-bracket sequences for all structures including pseudoknots.

The SPOT-RNA program was developed and validated using bpRNA data and can be usefully employed to improve RNA structure modelling, sequence alignment, and functional annotation. SPOT-RNA, using an open server and standalone software, improved the Matthews correlation coefficient and F1 score by approximately 10%, compared to the existing suboptimal solution.

#### 4.2.2. Machine/Deep Learning for the Prediction of 3D Aptamer Structure

For protein design problems, advances in the field of deep generative models have led to powerful approaches such as AlphaFold. The 3D structure of proteins can be predicted with high accuracy regardless of the homology of the sequences. AlphaFold can calculate the distances between residues and the predicted potential forces between residues are used for structuring the protein. A deep learning method can be applied to predict 3D genome folding for the optimization of the 3D structure of DNA. Akita, as one example, predicts genome folding with a deep CNN [114]. 

Previous modelling approaches for 3D genome folding cannot easily predict DNA mutations as they rely on epigenetic information as input, and genome folding is greatly affected by small algorithmic differences. Akita is a CNN that can predict genome folding using only DNA sequences. It is a method that takes a DNA sequence as an input and converts it into a predicted locus-specific genome folding.

**Figure 2:**
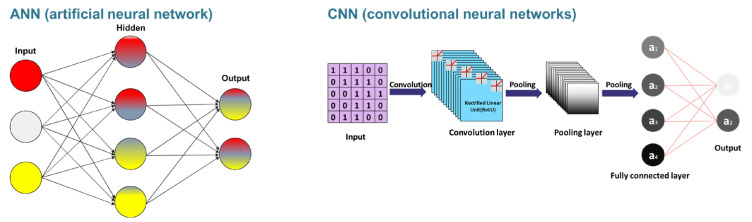
Conceptual diagram of ANN and CNN.

Akita can directly quantify the structural effects of nucleic acid sequence differences through in silico mutagenesis [114]. With Akita, the CNN can accurately predict genome folding using only DNA sequences (Figure 2). Akita can directly quantify nucleotide effects through in silico mutagenesis. After inducing mutations to specific motifs, the effect on genome folding was confirmed by predicting locus-specific patterns that were changed.

Mouse genome folding was predicted using a human-trained model (hESC output) using mouse DNA sequences as input using Akita, and genetically engineered inversions were predicted using a mouse-trained model (mESC output). These results confirm that nucleic acid structure prediction can be used in new organism conditions (mouse instead of human) and structural variation conditions (inversion instead of deletion), raising the possibility of cross-species analysis of genome folding.

Unlike previous machine learning methods, Akita can predict the effects of DNA mutations and characteristics of genome folding. Akita consists of a “trunk” and “head” based on baseji. The head has the function of learning DNA motifs with grammars combined in genome folding and recognising feature relationships. However, Akita can only reveal DNA genome folding and is not sufficient for predicting the details of the 3D structure of DNA aptamers. However, this method shows the potential of deep learning in predicting the 3D structure of DNA aptamers.

### 4.3. Trait-Based Machine Learning

Supervised machine learning consists of learning a function from labelled training data, and through this learning, it can be used to predict the outcomes of unlabelled data. The ability of aptamers to bind can be predicted through supervised machine learning.

Li et al. proposed a method of integrating features derived from aptamers and their target proteins in Aptamer Base, using the maximum relevance minimum redundancy (mRMR) method and incremental feature selection (IFS) method to select features, in which a random forest model was developed [115].

mRMR is a variable selection algorithm based on mutual information between entities. Calculated based on the classification class and feature values of entities, variables with higher relevance and smaller redundancy can be selected to indicate higher priority [116].

In IFS, feature selection is presented as a method to solve a problem called the ‘curse of dimensionality’. This refers to a problem in which the number of features increases exponentially as the number of objects increases, and feature selection refers to the process of extracting the features that can best represent a category [117].

The random forest model is a method of obtaining a conclusion by collecting classification results from a plurality of trees constructed through training. The average predicted value is derived from the decision tree in the learning process, and it is characterised by being able to overcome the overfitting limit of the decision tree [118]. 

Zhu et al. developed a text classification model to predict the interaction of the aptamer and target protein using the sequence characteristics extracted from aptamers and target proteins [119]. In this model, after the characterization of the feature of the target protein using a sparse autoencoder, the best combination of sequence characters was selected using the gradient boosting decision tree (GBDT) and incremental feature selection (IFS) methods. 

GBDT is one of the ensemble methodologies of predictive models, belonging to the boosting family, and is a supervised learning algorithm that uses gradients. Boosting refers to making a strong classifier by combining weak classifiers, and it is a method of continuously increasing the expressiveness of the model by using an additional model for data that did not fit the existing model well. Specifically, after sequentially fitting a new model that complements the weaknesses of the previous models, it goes through the process of generating a model obtained through linear combination of the previous models [120]. A prediction model was constructed based on three sub-support vector machine (SVM) classifiers. SVM is one of the supervised learning models used for pattern recognition and data analysis. It is mainly used for classification and regression analysis. Considering data divided into two sets, it is an algorithm that creates a non-probabilistic binary linear classification model that determines which set new data belong to. Unlike other algorithms with similar functions, it can relatively reduce learning data and is known to have high search accuracy. It is usefully employed in the medical field to separate up to 90% of proteins from classified compounds. Apta-LoopEnc [101] can design new aptamers using the SVM model. Apta-LoopEnc labels the candidates with high and low binding affinity. Nonetheless, these models require extensive training as they are based on experience and knowledge. Additionally, shallow machine learning models based on sequence data are usually unable to fully learn key features (e.g., distance correlation), which can result in inaccurate predictions. 

### 4.4. Deep Learnings for Developing Aptamers

Deep learning models can outperform machine learning models because they can model interactions between large numbers of atoms by learning features without feature engineering [121]. Deep learning has two uses: representation of input data and deep learning architecture.

In terms of input data, the aptamer–target binding affinity can be predicted by separating the input data into a sequence-based model and a structure-based model [10]. Meanwhile, deep learning architectures widely used in aptamer research are based on recurrent neural networks (RNNs), convolutional neural networks (CNNs), or general regression neural networks (GRNNs). RNN processes the sequence information as inputs. GRNN which is a variation to radial basis neural networks, trains samples that are averaged over the radial basis neuron. CNN is trained with convolutional layers and is a tool for pattern recognition; thus, the CNN can be used for the prediction of structural information [122].

Despite the power and accuracy of deep learning models for predicting aptamer binding ability, few cases have been reported so far. 

**Figure 3:**
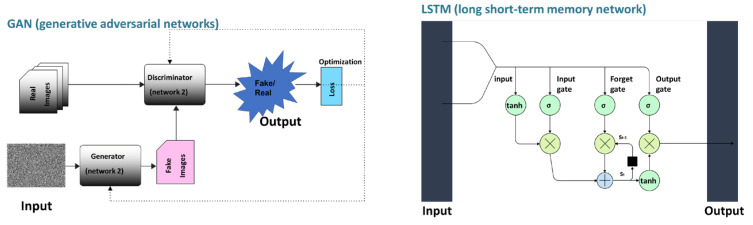
Conceptual diagram of GAN and LSTM.

Michael et al. applied a conditional variational autoencoder (CVAE) model for aptamers to the small molecule daunomycin to illustrate aptamer avidity [121]. The CAVE model uses a bidirectional long short-term memory network (LSTM), an RNN-based method, as an encoder, and a series of parallel feed-forward networks as a decoder, which allows the model to predict new aptamer sequences with high affinity without inferring structural data (Figure 3). In this manner, complex relationships of aptamer sequences can be predicted.

The machine-learning-based method for the prediction of binding affinity between the aptamer and influenza was developed by Yu et al. [123]. The structural feature of aptamer sequences was extracted using QSAR based on GRNN. The study proved the feasibility of a deep learning model as a tool for the prediction of the binding affinity and design of aptamer candidates.

Emami et al. developed AptaNet, which is a predictor based on a deep neural network [107]. AptaNet predicts the affinity of aptamer–protein using a multi-layer perceptron as a classification model.

## 5. Application of Machine/Deep Learning for Aptamer Prediction

In the case of machine learning, there are some models applied to predict the binding affinity of small-molecule drugs, which can be used to predict the affinity of aptamers. The most representative tools are Kronecker regularised least squares (KronRLS) and SimBoost, based on the hypothesis that similar drugs tend to have similar targets [100]. KronRLS creates Kronecker results of drugs and targets and uses various types of drugs and protein–protein similarity score matrices. 

SimBoost is a non-linear method for predicting drug–target binding affinity using gradient-boosting regression trees. Both the similarity matrix and the generated shape are used in this model. Compared to simple clustering methods, KronRLS utilises a real formula in drug target prediction. Therefore, it can better reflect the actual complexity of the drug target prediction problem in real applications. The regularised least squares approach (RLS) has been used in many applications [124]. SimBoost overcomes the limitations of linear dependence of drug–target binding. SimBoost also applies confidence scores to predictions because of bias in the training data set. In aptamer binding affinity prediction, an RLS model or a gradient-boosting regression tree can be applied.

For the prediction of the binding affinity between a drug and a target, Ashtawy et al. developed a machine learning-based scoring function, which integrates six regression methods: multiple linear regression, multivariate adaptive regression spline, k-nearest neighbour, SVM, random forest, and boosted regression tree [125]. The integrated machine learning model outperformed other single models as the adjusted parameter with a proper value obtained from cross-validation was used for prediction. The more multiple models combined, the higher the accuracy that can be obtained for the prediction of the binding affinity.

The deep learning model used in the development of small-molecule drugs can also be used in aptamer research to increase the accuracy and utilisation of aptamer development. The CNN-based Pafnucy algorithm extracts structural information, including 3D grid and 4D tensor information [126]. Prediction is possible through the AK-score applying a 3D CNN model [127]. The AK score is a new neural network model consisting of multi-channel 3D CNN layers that uses an ensemble of multiple independently trained networks to quickly and accurately predict the binding ability of a specific ligand to a target protein.

The artificial neural network (ANN)-based ensemble technique is a method that can be applied simply without changing the network structure. It is one of the most powerful tools for predicting avidity by combining properties from individual proteins and ligand structures.

The advantage of the ANN-based ensemble technique, compared to single neural network technology, is that it does not require modification of the network architecture and can be used in combination with existing methods. Ashtawy et al. found that the ensemble neural network scoring function was 19% more accurate in drug development than when using a single neural network [128]. DeepAffinity uses both CNN-based and RNN-based techniques [129]. When inputting protein sequences, an RNN-based Seq2seq model was used, which modelled sequence information through natural language processing. Then, features were trained using a CNN-based model. The attention mechanism was used to predict specific parts, drugs, and proteins. Word-based CNN models can represent sequence information. Word-based techniques characterise sequences and identify short residues that characterise proteins. This is an advantage compared to the character-based method. DeepDTA [130], WideDTA [131], CSatDTA [132], DeepMHADTA [133], and tranDTA [134] are used to predict the binding affinity between a drug and a target using CNN. WideDTA is a word-based method, whereas DeepDTA is character-based. WideDTA integrates information regarding protein sequences, ligand sequences, motifs, domain sequences of proteins, and maximum common substructure binding to ligands, which consist of four textual units. As this provides more information, the accuracy is higher than the method of analysing only the protein–ligand sequence. Thus, WideDTA shows improved performance compared to DeepDTA.

Methods based on generative adversarial networks (GANs) can handle large databases. GANsDTA [135] is a semi-supervised learning method that generates fake samples using a given noise distribution. Afterwards, fake and real samples are used for classification. Consequently, as it is a GAN-based method, it shows similar performance to DeepDTA and shows better performance when large databases are used.

Deep learning models use neural networks to learn from large amounts of data, like the human brain.

Both are composed of an input layer and an output layer, and ANN has a hidden layer between the input layer and the output layer. Every node in one layer is connected to a node in the next layer. GAN consists of a generator model that generates new data and a discriminator model that identifies whether input data is real data, domain-derived data, or fake data. RNN-based LSTM networks consist of an input gate, output gate, and forget gate. LSTM calculates the model‘s memory and input weights using input values from previous timesteps. CNN consists of convolution layers with filters, pooling layers, fully connected layers, and softmax functions. CNNs are widely used in image retrieval.

## 6. Conclusions

In computer-aided drug design, structure-based methods are most often used. In drug design, the binding affinity between a target and ligand has been predicted using machine-learning- or deep-learning-based techniques. Various models can be applied to machine learning or deep learning to predict aptamer binding. In addition, the binding affinity between the aptamer and target can be predicted using a structure-based machine learning or deep learning method. Docking, molecular dynamics, quantum–chemical calculations, and QSAR can be used for in silico aptamer design. QSAR and machine learning can inform aptamer design and modelling. The binding affinity and stability of aptamers designed in silico are also verified by in vitro methods. 

With the rapid evolution of computing power or NGS technology, the information of the sequence is becoming increasingly huge. According to this flow, it is necessary to build a data bank of SELEX. One example may be a constructing verified dataset obtained by specific experimental results with different modeling methods. The case study should be conducted through the comparison of binding affinity calculated by various modeling methods with the actual experimental result data. In addition, when conducting HT-SELEX, instead of analyzing the final pool, all of the pool can be analyzed for the rich data set because the candidates from the final round do not always produce the best candidate aptamer. Moreover, experimental data on the sequence predicted through modeling, or the vast sequence data obtained through HT-SELEX, should be gathered and constructed as a dataset. Because of insufficient accumulated data for the prediction of the aptamer, the data required for AI training are also not enough. Only a few datasets are labeled correctly, which affects the performance of AI-based models. These reliable datasets can be used as a criterion for building modeling techniques as well as acting as training data for machine learning.

In addition, in the aptamer study, the experimental stability of the aptamer is improved by modifying a 2′ or a terminal with cholesterol or polyethylene glycol. In predicting the secondary or tertiary structure of aptamer, it is necessary to develop a tool that can reflect the influence of such modification.

Through this review paper, it is expected that a high-throughput, in silico aptamer development method can be developed and used for aptamer screening and characterization.

## Figures and Tables

**Table 1:** Comparison of the dissociation constant of the aptamer after in silico process.

Target	Before In Silico Method	In Silico Method	References
Aflatoxin B1	38.5 pM	4.02 pM	[95]
EpCAM	39.89 nM	10.78 nM	[82]
Vascular Endothelial Growth Factor	200 nM	52 nM	[96]
Vascular Endothelial Growth Factor	4.7 nM	300 pM	[97]

**Table 2:** In silico tools for designing aptamers.

Tools	Features	Reference
Apta-loopEnc	Labels the candidates with high and low binding affinity. Predicts aptamer based on SVM	[101]
AptaSUITE	Framework analysis of data from HT-SELEX such as sequences and aptamer counts.	[102]
SMART-Aptamer	Predicts aptamers based on ranking of sequence abundance, stability of the secondary structure	[77]
RaptRanker	Predicts aptamer based on structure and frequency of sequence	[103]
PPAI (http://39.96.85.9/PPAI/, accessed on 30 December 2022)	Web server for prediction of aptamers and interaction between protein and aptamer	[104]
AptCompare	Meta-analysis platform for HT-SELEX	[105]
APTANI^2^	GUI platform for aptamers based on frequency of sequence and stability of secondary structure	[106]
AptaNet	Predicts the affinity of aptamer-protein using a multi-layer perceptron as a classification model.	[107]
